# Effectiveness of a Web-Based Tailored Intervention (E-health4Uth) and Consultation to Promote Adolescents’ Health: Randomized Controlled Trial

**DOI:** 10.2196/jmir.3163

**Published:** 2014-05-30

**Authors:** Rienke Bannink, Suzanne Broeren, Evelien Joosten-van Zwanenburg, Els van As, Petra van de Looij-Jansen, Hein Raat

**Affiliations:** ^1^Erasmus University Medical Center RotterdamDepartment of Public HealthRotterdamNetherlands; ^2^Regional Public Health & Youth Service South-Holland SouthDordrechtNetherlands; ^3^Consortium Rivas-CareynDepartment of Youth Health CareGorinchemNetherlands; ^4^Municipal Public Health Service Rotterdam areaRotterdamNetherlands

**Keywords:** adolescents, youth health care, eHealth, Internet, Web-based tailoring, consultation, randomized controlled trial, health promotion, prevention

## Abstract

**Background:**

To promote well-being and health behaviors among adolescents, 2 interventions were implemented at 12 secondary schools. Adolescents in the E-health4Uth group received Web-based tailored messages focused on their health behaviors and well-being. Adolescents in the E-health4Uth and consultation group received the same tailored messages, but were subsequently referred to a school nurse for a consultation if they were at risk of mental health problems.

**Objective:**

This study evaluated the effect of E-health4Uth and E-health4Uth and consultation on well-being (ie, mental health status and health-related quality of life) and health behaviors (ie, alcohol and drug use, smoking, safe sex).

**Methods:**

A cluster randomized controlled trial was conducted among third- and fourth-year secondary school students (mean age 15.9, SD 0.69). School classes (clusters) were randomly assigned to (1) E-health4Uth group, (2) E-health4Uth and consultation group, or (3) control group (ie, care as usual). Adolescents completed a questionnaire at baseline and at 4-month follow-up assessing alcohol consumption, smoking, drug use, condom use, mental health via the Strengths and Difficulties Questionnaire (SDQ) and the Youth Self Report (YSR; only measured at follow-up), and health-related quality of life. Multilevel logistic, ordinal, and linear regression analyses were used to reveal differences in health behavior and well-being between the intervention groups and the control group at follow-up. Subsequently, it was explored whether demographics moderated the effects.

**Results:**

Data from 1256 adolescents were analyzed. Compared to the control intervention, the E-health4Uth intervention, as a standalone intervention, showed minor positive results in health-related quality of life (B=2.79, 95% CI 0.72-4.87) and condom use during intercourse among adolescents of Dutch ethnicity (OR 3.59, 95% CI 1.71-7.55) not replicated in the E-health4Uth and consultation group. The E-health4Uth and consultation intervention showed minor positive results in the mental health status of adolescents (SDQ: B=−0.60, 95% CI −1.17 to −0.04), but a negative effect on drug use among boys (OR 0.36, 95% CI 0.13-0.96). In the subgroup of adolescents who were at risk of mental health problems at baseline (and referred for a consultation with the nurse), the E-health4Uth and consultation group showed minor to moderate positive results in mental health status (SDQ: B=−1.79, 95% CI −3.35 to −0.22; YSR: B=−9.11, 95% CI −17.52 to −0.71) and health-related quality of life (B=7.81, 95% CI 2.41-13.21) at follow-up compared to adolescents in the control group who were at risk of mental health problems at baseline.

**Conclusions:**

Findings from this study support the use of the E-health4Uth and consultation intervention in promoting the well-being of adolescents at risk of mental health problems. Future research is needed to further evaluate the effects of the consultation as a standalone intervention, and the dual approach of further tailored eHealth messages and a consultation.

**Trial Registration:**

Nederlands Trial Register: NTR 3596; http://www.trialregister.nl/trialreg/admin/rctview.asp?TC=3596 (Archived by WebCite at http://www.webcitation.org/6PmgrPOuv).

## Introduction

### Background

A high percentage of adolescents suffer from mental health problems, and many health-risk behaviors, such as excessive alcohol consumption, cigarette smoking, use of drugs, and having unsafe sex, are acquired during adolescence [1]. These mental health problems and health-risk behaviors often persist into adulthood, thereby affecting not only current health but also health later in life [2-4]. Given this, reducing the burden of adolescent mental health problems and health-risk behaviors is a major public health priority, one in which preventive youth health care can play an important role.

Many countries have established preventive youth health care, which refers to a variety of activities aimed at improving and protecting the health, growth, and development of young people. These activities include a system of child health care, which serves children from birth through to 18 years. In the Netherlands, all children and adolescents are invited by youth health care organizations to attend regularly scheduled preventive health consultations until the age of 13 years [5]. These consultations with a nurse or physician focus on growth, development, health functioning, and behaviors of infants, children, and adolescents. Furthermore, the consultations are funded by the government, are free of charge, and take place at the preventive youth health care center or at school. Given the rapid rate of maturation in adolescence and the mental health problems and health-risk behaviors associated with this developmental period, the government in the Netherlands encourages an additional preventive health consultation at age 15-16 years [6].

Previous research shows the use of Web-based applications for delivering tailored preventive messages in current preventive youth health care practice to be a promising development [7-9]. Web-based tailoring is a health education technique that enables the adaptation of information to individual characteristics. Web-based tailored messages eliminate (as far as possible) information that is not personally relevant [10,11] and are, therefore, more likely to be effective in changing behavior compared to nontailored messages [11]. Additionally, they facilitate the enhanced efficiency of face-to-face consultations by collecting information on adolescents’ health before the consultation, which a professional can use during the consultation [7,12,13].

To promote well-being and health behaviors among adolescents, 2 interventions using Web-based tailored messages (E-health4Uth and E-health4Uth and consultation) were implemented in a preventive youth health care setting. The Web-based tailored messages focused on topics related to health-risk behaviors (eg, alcohol consumption, smoking) and well-being (eg, mental health status, suicidal thoughts). Both interventions used the same Web-based tailored messages, which were developed for adolescents (aged 12-18 years) in an earlier study [14]. In the E-health4Uth and consultation group, adolescents who were at risk of mental health problems were also referred to a school nurse for a consultation. Consequently, the intervention in this subgroup was more extensive. To facilitate communication during the consultations [7], the nurses received information regarding the adolescents’ well-being and health behaviors from the E-health4Uth tool, with the adolescents’ knowledge. A first investigation showed that the Web-based tailored messages and additional consultation were positively experienced by the adolescents and nurses alike [15]. However, the effectiveness of these interventions are currently unknown.

### Objective of the Study

This study evaluates the effect of E-health4Uth and E-health4Uth and consultation on well-being (ie, mental health status and health-related quality of life) and health behaviors (ie, alcohol and drug use, smoking, safe sex) as applied by preventive youth health care in secondary schools. The hypotheses of the study are twofold. First, it is expected that adolescents in the E-health4Uth group will show a higher level of well-being and less risky behavior at 4-month follow-up compared to the control group (ie, care as usual). Second, it is expected that adolescents in the E-health4Uth and consultation group will show a higher level of well-being and less risky behavior (alcohol and drug use, smoking, safe sex) at 4-month follow-up compared to the control group (ie, care as usual). In addition, to gain more insight into the combined effect of E-health4Uth with a consultation, we assessed effects on well-being in the subgroup of adolescents’ at risk of mental health problems at baseline, because only these adolescents were invited for a consultation with the nurse.

## Methods

### Study Design

A 3-armed cluster randomized controlled trial (RCT) was conducted from September 2012 to May 2013 with measurements at baseline and 4 months after the baseline measurement (trial registration: Current Controlled Trials NTR3596). The interventions were applied by preventive youth health care in secondary schools. School classes (clusters) were randomly assigned to one of the study arms (ie, E-health4Uth, E-health4Uth and consultation, control group). School classes were the unit of randomization because randomization at the individual level (ie, the level of the adolescents) can lead to contamination of the control group [16]. A computer-generated list of random numbers was used to allocate the school classes (clusters) to one of the study arms. The randomization sequence was stratified with a 1:1:1 allocation using random block sizes of 3. This list was prepared by an investigator with no involvement in the trial and was applied by the researchers. The research proposal was reviewed by the Daily Board of the Medical Ethical Committee of Erasmus MC. As a result of this review, the Committee declared that the Medical Research Involving Human Subjects Act (also known by its Dutch abbreviation WMO) did not apply to this research proposal. The Medical Ethical Committee had no objection to the execution of this research proposal (MEC-2012-337). Further details about the study design and the interventions are provided in a design paper published elsewhere [17].

### Participants and Procedures

Two youth health care organizations in the Dutch cities of Dordrecht and Zwijndrecht participated in this study and conducted the interventions in secondary schools. Of the 14 secondary schools invited by the youth health care organizations to participate in the study, 12 agreed and provided a total of 11 classes of third-year students (2 schools) and 75 classes of fourth-year students (10 schools). In the Netherlands, adolescents in the third and fourth years of secondary school are on average 15-16 years of age.

**Figure 1:**
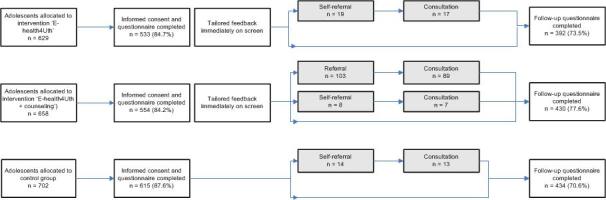
Flowchart of adolescents’ participation.

A few weeks before the start of the study, all adolescents and parents received information about the study. If parents did not want their child to participate, they could object to their child’s participation. Adolescents were asked to provide written consent before they completed the baseline questionnaire. Of the 1989 eligible adolescents, 1702 (85.57%) participated: 533 (84.7%) in the E-health4Uth group, 554 (84.2%) in the E-health4Uth and consultation group, and 615 (87.6%) in the control group (Figure 1). The main reason for nonparticipation was absence, primarily because of illness. Furthermore, 29 parents refused their child’s participation and 24 adolescents refused participation themselves.

At 4-month follow-up, 3 schools did not schedule the follow-up classroom assessments for all or several classes (missing data from 14 classes). At the remaining schools, 135 adolescents were absent at follow-up. In total, 1256 adolescents participated at 4-month follow-up (73.79%): 392 of 533 in the E-health4Uth group (73.5%), 430 of 554 in the E-health4Uth and consultation group (77.6%), and 434 of 615 in the control group (70.6%).

### The E-health4Uth Intervention

During one classroom session (approximately 45 min), adolescents completed a self-report questionnaire via the Internet to assess health-risk behavior and well-being with respect to the following topics: alcohol consumption, drug use, smoking, sexual behavior, bullying, mental health status, suicidal thoughts, suicide attempts, and unpleasant sexual experiences (Multimedia Appendix 1). This questionnaire served as a basis to tailor the messages, but also as a baseline measurement for the effect evaluation. The questionnaire was constructed based on several existing instruments used by municipal public health services and health institutes [18]. Consensus on the use of these instruments was established by the National Institute for Public Health and Environment (RIVM), the Dutch association for residential and homecare organizations and infant and child health clinics (Actiz), and the Association of Municipal Public Health Services in the Netherlands (GGD Nederland).

**Figure 2:**
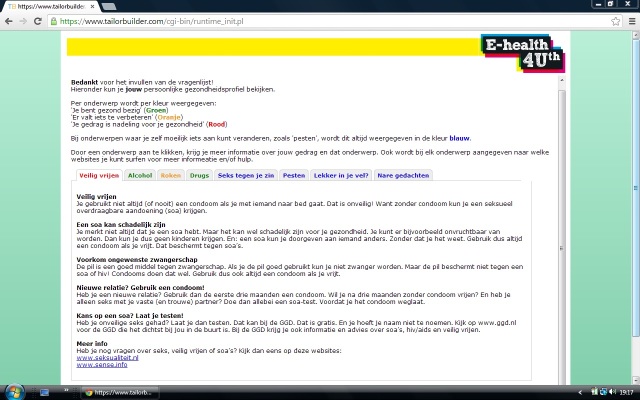
Screenshot of the Web-based tailored messages. This message was presented to adolescents who answered that they have had unsafe sex (left-most tab). The message is displayed in red, indicating unhealthy behavior. The messages on the other topics are presented when clicking on the other (colored) tabs.

After completing the questionnaire, the participants were presented with a message of approximately the same length for each topic (see Figure 2 for an example of a message on one topic). We used Web-based tailored messages that were developed for adolescents (aged 12-18 years) and applied in an earlier study [14]. The messages were developed by the Department of Health Promotion and Health Education of the University of Maastricht. The messages were tailored to the answers given in the questionnaire. Tailored feedback is more useful in motivating people to perform the desired behaviors than nontailored feedback [11]. It also provides the opportunity to give normative feedback (ie a comparison between individual responses and the health norms) and positive feedback to reinforce desired states, both of which were used in this study.

For each topic, a score was computed which was compared with the Dutch health norms for adolescents [14,18,19]. Based on this score, a message was immediately presented on the screen that reflected the person’s current behavior or well-being in relation to the Dutch health norm, and the adolescent was offered advice to change unhealthy behavior and/or to talk to a person of trust (Figure 2). The messages were displayed in red, orange, or green, indicating unhealthy behavior, behavior just below the norm, or behavior meeting the Dutch health norm, respectively. The topics on well-being were always displayed in blue.

With links to relevant websites, adolescents were encouraged to read more information on the topics. At the end of the program, adolescents were invited to follow the Facebook page of E-health4Uth to find more information on the topics. Additionally, adolescents could check a box for a self-referral to the nurse or could send an email to the nurse. After 1 month, adolescents received a reminder of the tailored messages by email.

### The E-health4Uth and Consultation Intervention

During a classroom session, adolescents in the E-health4Uth and consultation group completed the same questionnaire and received the same intervention as that applied in the E-health4Uth--only group. Additionally, adolescents at risk of mental health problems were invited for a consultation with the nurse. Adolescents were classified as at risk of mental health problems when their score on the total problem scale of the Strengths and Difficulties Questionnaire (SDQ) was higher than 16, and/or their score on the SDQ for emotional problems was higher than 5, and/or they reported having suicidal thoughts occasionally or more frequently (or did not want to answer this question), and/or they reported a suicide attempt within the past year (or did not want to answer this question) [17].

The consultation took place at school and was provided by school nurses who were already working at the schools and who had already provided consultations to adolescents at approximately 13 years of age. These nurses were trained to apply motivational interviewing with adolescents at age 15-16 years. They received the results of the assessment for each referred adolescent before the consultation. During the consultation, the nurses focused on specific risk areas and on mental health in particular. Furthermore, they either initiated a further consultation with themselves or referred adolescents to another professional if they deemed this necessary.

### Control Group

Adolescents in the control group completed the same questionnaire assessing health-risk behaviors and well-being as adolescents in the intervention groups, with the exception of the questions on unpleasant sexual experience, suicidal thoughts, and suicidal attempts, because these questions were used only to tailor the messages in the intervention group, not as measurements to assess the effectiveness of the interventions [17]. Adolescents in the control group received no messages afterwards based on their scores.

### Measures

#### Overview

The primary outcomes of the study were adolescents’ health behaviors (ie, alcohol and drug use, smoking, safe sex) and mental health status. The secondary outcome of the study was health-related quality of life. The self-report questionnaire, used to tailor the eHealth messages in the 2 intervention groups, also served as the baseline questionnaire.

#### Health Behaviors

The questionnaire used to assess health behaviors was based on existing instruments previously developed by municipal public health services and health institutes in the Netherlands [18]. This questionnaire was administered at baseline and at 4-month follow-up. In this questionnaire, the frequency of alcohol consumption, smoking, drug use, and condom use were assessed on ordinal scales. Alcohol consumption was covered by 2 items: (1) how often did you drink 5 or more drinks on 1 occasion in the past 4 weeks (never to 9 or more times), and (2) how often have you been drunk or tipsy in the past 4 weeks (never to 20 or more times).

Drugs use was assessed by how often the adolescent had used drugs in the past 4 weeks (never to 20 or more times), smoking by how often the adolescent currently smokes (not at all to every day), and condom use by how often the adolescent had used condoms during intercourse (never to always). This last question was only presented if it was applicable (ie, when an adolescent had answered he/she was sexually active).

#### Well-Being

Mental health status was assessed by the Strengths and Difficulties Questionnaire (SDQ) [20,21] and the Youth Self Report (YSR) [22]. The SDQ consists of 25 items describing positive and negative attributes of adolescents that can be allocated to 5 subscales of 5 items each: the emotional problems, the conduct problems, the hyperactivity-inattention, the peer problems, and the prosocial behavior subscales. Each item is scored on a 3-point scale (0=not true, 1=somewhat true, and 2=certainly true). A total difficulties score is calculated by summing the scores for the emotional problems, conduct problems, hyperactivity-inattention, and peer problems subscales (range 0-40). The YSR comprises 119 items addressing emotional and behavioral problems of adolescents. Respondents have to indicate on 3-point scales to which extent each item applies to him/her (0=not, 1=sometimes, or 2=often). A total score is calculated by summing the scores on all items (range 0-210).

Health-related quality of life was measured at baseline and at 4-month follow-up by 4 items of the general health perceptions scale of the Child Health Questionnaire-Child Form (CHQ-CF-GH4) [23]. One item is scored on a 5-point scale of 1=excellent, 2=very good, 3=good, 4=moderate, 5=bad and 3 items are scored on a 5-point scale of 1=true, 2=usually true, 3=do not know, 4=usually not true, 5=not true. A total score is calculated by weighing the scores and summing the weighed scores for all items (range 0-100).

The SDQ and CHQ-CF-GH4 were administered at baseline and at 4-month follow-up. The YSR was administered only at 4-month follow-up to reduce respondent burden at baseline. At baseline, adolescents also received the E-health4Uth messages after the questionnaires.

#### Demographics

Age (assessed by date of birth), gender, country of birth of the adolescent and both parents, and the level of education that the adolescents attended were assessed in the baseline questionnaire. In the Netherlands, a distinction is made in the levels of education adolescents attend at secondary schools. Lower levels of education are referred to as vocational training and higher levels of education are referred to as preuniversity education. Ethnicity was classified as Dutch or non-Dutch, in accordance with the definitions of Statistics Netherlands [24]; adolescents with at least 1 parent born outside the Netherlands were classified as non-Dutch.

### Statistical Analysis

Descriptive statistics were used to describe the characteristics of adolescents in the 3 study conditions. Differences between each of the intervention conditions and the control condition, as measured at baseline, were tested with independent sample *t* tests (continuous variables), Mann-Whitney *U* tests (ordinal variables), and chi-square tests (categorical variables). The effectiveness of E-health4Uth and E-health4Uth and consultation was investigated by means of multilevel logistic (categorical variables), ordinal (ordinal variable), and linear (continuous variables) regression analyses. Multilevel analysis adjusts for clusters (ie, classes) by taking the dependency between observations of adolescents from the same class into account. For the multilevel linear regression analyses, a bootstrapping method was used [25]. This method deals with data that are skewed, as is often the case with data on well-being and in this study. All regression analyses were adjusted for demographic factors that significantly differed between each of the intervention conditions and the control condition. All regression analyses were also adjusted for the baseline value of each outcome, with the exception of the YSR because this questionnaire was only assessed at follow-up. Therefore, the results of the YSR analyses were only adjusted for demographic factors that significantly differed between each of the intervention conditions and the control condition. However, exploratory analyses showed that when adjusting for the baseline value of the SDQ, which assesses a similar concept (mental health) as the YSR [21], similar results were obtained in the YSR analyses as when not adjusting for the SDQ baseline value.

To gain more insight into the combined effect of E-health4Uth with a consultation, we tested the effects on well-being in the subgroup of adolescents at risk of mental health problems at baseline because only these adolescents were referred for a consultation with the nurse. The effectiveness was tested by means of multilevel linear regression analyses (with a bootstrap procedure). The subgroup consisted of those with a score on the SDQ of >16 or a score of >5 on the emotional problems subscale of the SDQ [17]. Suicidal thoughts and suicide attempts could not be used to classify adolescents at risk of mental health problems at baseline because suicidal thoughts and suicide attempts were not assessed in the control group. These questions were not administered in this group because this group did not receive an intervention in which these concerns were addressed.

Subsequently, it was explored whether gender, ethnicity, or level of education moderated the effects of E-health4Uth and E-health4Uth and consultation on health behaviors and well-being. This was done by adding an intervention dummy × demographic factor interaction term to the regression analyses. If the interaction term was significant at *P*<.05, a stratified analysis was conducted.

Participants were analyzed in the groups to which they had been randomized, regardless of whether they received the allocated intervention or not (eg, not attending consultation after an invitation). Each analysis of the effectiveness of the intervention was performed on the follow-up data that was available on the outcome concerned. The multilevel regression analyses were performed in Stata 13.0 (StataCorp LP, College Station, TX, USA). Other analyses were performed in SPSS 21.0 (IBM Corp, Armonk, NY, USA). The significance level was set at .05 and tests were 2-sided. To indicate the clinical significance of any benefits of the interventions, we also report odds ratios (OR) for categorical and ordinal outcomes and Cohen’s *d* (*d*) for continuous outcomes.

## Results

### Nonresponse Analysis

Chi-square tests and *t* tests were conducted to compare adolescents participating at follow-up with adolescents not participating at follow-up. Participating at follow-up (yes/no) was used as the dependent variable and gender, age, education, ethnicity, and study condition as independent variables. Group differences were found for gender, age, education, ethnicity, and study condition, with the adolescents not participating at follow-up more often being female (χ^2^_1_=4.1, *P*=.04), older (*t*_*680*_=6.69, *P*<.001), lower educated (χ^2^_1_=20.0, *P*<.001), of non-Dutch ethnicity (χ^2^_1_=64.7, *P*<.001), and allocated to the control group instead of the E-health4Uth and consultation group (χ^2^_1_=7.5, *P*=.006).

### Adolescents’ Characteristics

The average age of the adolescents in this study was 15.9 years (SD 0.69); 54.70% (687/1256) of the sample consisted of boys, 76.19% (957/1256 were of Dutch ethnicity, 50.48% (634/1256) attended vocational training, and 49.52% (622/1256) preuniversity education. Table 1 shows general characteristics and baseline health behaviors and well-being of adolescents in the 3 study conditions. At baseline, a lower percentage of adolescents in the E-health4Uth group had used drugs in the past 4 weeks compared to adolescents in the control group (4.6% vs 8.1%; *P*<.04). Further, adolescents in the E-health4Uth and consultation group were significantly younger than adolescents in the control group (mean 15.95, SD 0.70 vs mean 15.79, SD 0.66; *P*<.001). Therefore, all analyses evaluating the effectiveness of E-health4Uth and consultation were adjusted for age.

**Table 1:** General characteristics and baseline health behaviors and well-being of adolescents for the intervention groups and control group (N=1256).

Characteristics			E-health4Uth n=392	E-health4Uth + consult n=430	Control n=434	*P* value	
						E-health4Uth vs control	E-health4Uth + consult vs control
Number of school classes			27	26	25		
Age (years), mean (SD)			15.84 (0.70)	15.95 (0.70)	15.79 (0.66)	.28	<.001^a^
**Gender, n (%)**							
	Male		223 (56.9)	241 (56.0)	223 (51.4)	.11	.17^b^
	Female		169 (43.1)	189 (44.0)	211 (48.6)		
**Ethnicity, n (%)**							
	Dutch		311 (79.3)	320 (74.4)	326 (75.1)	.15	.81^b^
	Non-Dutch		81 (20.7)	110 (25.6)	108 (24.9)		
**Educational level, n (%)**							
	Vocational training		191 (48.7)	231 (53.7)	212 (48.8)	.97	.15^b^
	Preuniversity		201 (51.3)	199 (46.3)	222 (51.2)		
**Alcohol consumption**							
	**5 or more drinks on 1 occasion in the past 4 weeks, n (%)**						
		0 times	255 (65.1)	272 (63.4)	292 (67.6)	.48^c^	.20^c^
		1 times	962 (15.8)	69 (16.1)	62 (14.4)		
		2 times	36 (9.2)	36 (8.4)	34 (7.9)		
		3-4 times	22 (5.6)	35 (8.2)	29 (6.7)		
		5 or more times	17 (4.3)	17 (4.0)	15 (3.5)		
	**Have been drunk or tipsy in the past 4 weeks, n (%)**						
		0 times	290 (74.0)	318 (74.1)	333 (77.1)	.28^c^	.30^c^
		1 times	54 (13.8)	60 (14.0)	53 (12.3)		
		2 times	21 (5.4)	22 (5.1)	24 (5.6)		
		3 or more times	27 (6.9)	29 (6.8)	22 (5.1)		
**Smoking, n (%)**							
	No		329 (83.9)	352 (82.1)	352 (81.5)	.39^c^	.92^c^
	Less than once a week		13 (3.3)	16 (3.7)	19 (4.4)		
	At least once a week, but not every day		15 (3.8)	14 (3.3)	20 (4.6)		
	Every day		35 (8.9)	47 (11.0)	41 (9.5)		
**Drug use (past 4 weeks), n (%)**							
	0 times		274 (95.4)	402 (93.7)	397 (91.9)	.04^b^	.31^b^
	1 or more times		18 (4.6)	27 (6.3)	35 (8.1)		
**Condom use during intercourse (n=324), n (%)**							
	Always		52 (53.1)	68 (52.3)	49 (51.0)	.50^c^	.55^c^
	Usually		21 (21.4)	25 (19.2)	15 (15.6)		
	Sometimes/almost never		14 (14.3)	25 (19.2)	18 (18.8)		
	Never		11 (11.2)	12 (9.2)	14.6 (14)		
**Well-being, mean (SD)** ^d^							
	SDQ score		10.06 (5.57)	9.75 (5.14)	9.91 (5.32)	.69^a^	.67^a^
	CHQ-CF-GH4 score		71.39 (17.87)	71.62 (18.49)	73.67 (17.78)	.07^a^	.10^a^

^a^Independent-samples *t* test.

^b^Chi-square test.

^c^Mann-Whitney *U* test.

^d^SDQ: Strengths and Difficulties Questionnaire (higher score indicates more mental health problems; range 0-40); CHQ-CF-GH4: Child Health Questionnaire-Child Form-General Health (higher score indicates a better health-related quality of life; range 0-100).

**Table 2:** Follow-up health behaviors and well-being of adolescents and effects of the interventions with the control group as reference (N=1252).

Behaviors and well-being				E-health4Uth n=392	E-health4Uth + consult n=430	Control n=434	E-health4Uth vs control^a^		E-health4Uth + consult vs control group^b^	
							OR/B (95% CI)	*P*	OR/B (95% CI)	*P*
**Alcohol consumption, n (%)**										
		**5 or more drinks on 1 occasion in the past 4 weeks**								
			0 times	230 (59.0)	280 (65.9)	276 (63.7)	0.90 (0.61, 1.34)^c^	.62	1.21 (0.77, 1.26)^c^	.35
			1 times	62 (15.9)	44 (10.4)	58 (13.4)				
			2 times	43 (11.0)	32 (7.5)	37 (8.5)				
			3-4 times	28 (7.2)	46 (10.8)	34 (7.9)				
			5 or more times	27 (6.9)	23 (5.4)	28 (6.5)				
		**Have been drunk or tipsy in the past 4 weeks**								
			0 times	275 (70.5)	317 (74.6)	321 (74.1)	0.90 (0.61, 1.35)^c^	.62	1.22 (0.85, 1.74)^c^	.29
			1 times	57 (14.6)	52 (12.2)	57 (13.2)				
			2 times	18 (4.6)	20 (4.7)	20 (4.6)				
			3 or more times	40 (10.3)	36 (8.5)	35 (8.1)				
**Smoking, n (%)**										
	No			323 (82.8)	351 (82.6)	349 (80.8)	0.97 (0.61, 1.56)^c^	.90	0.95 (0.58, 1.57)^c^	.84
	Less than once a week			21 (5.4)	14 (3.3)	23 (5.3)				
	At least once a week, but not every day			14 (3.6)	11 (2.6)	19 (4.4)				
	Every day			32 (8.2)	49 (11.5)	41 (9.5)				
**Drug use (past 4 weeks), n (%)**										
	0 times			367 (94.1)	381 (89.6)	396 (91.7)	1.06 (0.43, 2.61)^d^	.90	0.65 (0.26, 1.59)^d^	.34
	1 or more times			23 (5.9)	44 (10.4)	36 (8.3)				
**Condom use during intercourse (n=376), n (%)**										
	Always			62 (52.1)	66 (43.7)	43 (40.6)	2.09 (1.04, 4.22)^c^	.04	1.36 (0.76, 2.44)^c^	.31
	Usually			24 (20.2)	32 (21.2)	15 (14.2)				
	Sometimes/almost never			18 (15.1)	38 (25.2)	27 (25.5)				
	Never			15 (12.6) (15)	15 (9.9)	21 (19.8)				
**Well-being, mean (SD)**										
	SDQ score^f^			8.92 (5.26)	8.42 (5.05)	9.07 (5.38)	−0.24 (−0.78, 0.29)^e^	.37	−0.60 (−1.17, −0.04)^e^	.04
	YSR score^f^			33.89 (23.02)	31.58 (22.58)	34.75 (25.26)	−0.89 (−4.18, 2.40)^e^	.60	−2.74 (−5.92, 0.44)^e^	.09
	CHQ-CF-GH4 score^g^			75.34 (16.56)	74.00 (18.49)	73.73 (18.17)	2.79 (0.72, 4.87)^e^	.008	1.03 (−1.12, 3.19)^e^	.35

^a^E-health4Uth vs control group: analyses were adjusted for the baseline value of each outcome.

^b^E-health4Uth and consultation vs control group: analyses were adjusted for age and the baseline value of each outcome.

^c^Multilevel ordinal regression; OR (95% CI).

^d^Multilevel logistic regression; OR (95% CI).

^e^Multilevel linear regression; Beta coefficient (95% CI).

^f^A higher score indicates more mental health problems (SDQ range 0-40, YSR range 0-210).

^g^A higher score indicates a better health-related quality of life (range 0-100).

### Effects of E-health4Uth

Adolescents in the E-health4Uth group used condoms significantly more often at follow-up compared to adolescents in the control group (52.1% vs 40.6%; OR 2.09, 95% CI 1.04-4.22) (Table 2). Furthermore, the health-related quality of life of adolescents in the E-health4Uth group was significantly better at follow-up compared to adolescents in the control group (mean 75.34, SD 16.56 vs mean 73.73, SD 18.17; B=2.79, 95% CI 0.72-4.87; *d*=0.09). No other effects of the E-health4Uth intervention on health behaviors or well-being were found.

### Effects of E-health4Uth and consultation

At follow-up, adolescents in the E-health4Uth and consultation group reported a significantly better mental health status compared to adolescents in the control group (SDQ: mean 8.42, SD 5.05 vs 9.07, SD 5.38; B=−0.60, 95% CI −1.17 to −0.04; *d*=0.12) (Table 2). No effects of the E-health4Uth and consultation intervention on health behaviors were found.

Adolescents in the E-health4Uth and consultation group, who were at risk of mental health problems at baseline and were therefore referred for a consultation with the nurse, reported a significantly better mental health status (SDQ: mean 12.79, SD 5.63 vs 14.57, SD 5.03; B=−1.79, 95% CI −3.35 to −0.22; *d*=0.33; YSR: mean 48.13, SD 25.45 vs 57.12, SD 27.66; B=−9.11, 95% CI −17.52 to −0.71; *d*=0.34) and a better health-related quality of life (mean 69.56, SD 18.37 vs 62.53, SD 20.08; B=7.81, 95% CI 2.41-13.21; *d*=0.37) at follow-up compared to adolescents in the control group who were at risk of mental health problems at baseline (Table 3). These results were not replicated among adolescents who were at risk of mental health problems in the E-health4Uth standalone intervention group (Table 3), indicating that the dual approach of advice and a consultation (ie, E-health4Uth and consultation) may have been responsible for the positive effects on well-being.

**Table 3:** Follow-up well-being of adolescents who were at risk of mental health problems at baseline and the effects of the interventions on well-being, with the control group as reference (n=194).

Well-being	Adolescents at risk of mental health problems, mean (SD)			E-health4Uth vs control^b^		E-health4Uth + consult vs control^c^	
	E-health4Uth^a^ n=63	E-health4Uth + consult^a^ n=63	Control^a^ n=68	Beta (95% CI)	*P*	Beta (95% CI)	*P*
SDQ score^d^	14.44 (5.67)	12.79 (5.63)	14.57 (5.03)	0.04 (−1.60, 1.68)	.96	−1.79 (−3.35, −0.22)	.03
YSR score^d^	56.49 (27.86)	48.13 (25.45)	57.12 (27.66)	−0.63 (−9.72, 8.47)	.89	−9.11 (−17.52, −0.71)	.03
CHQ-CF-GH4^e^	67.59 (17.14)	69.56 (18.37)	62.53 (20.08)	4.78 (−0.70, 10.25)	.09	7.81 (2.41, 13.21)	.005

^a^In the E-health4Uth (5 of 63) and control group (4 of 68), some adolescents at risk of mental health problems also attended the consultation after they referred themselves to the nurse. In the E-health4Uth and consultation group, 57 of the 63 referred adolescents attended the consultation.

^b^E-health4Uth vs control group: analyses were adjusted for the baseline value of each outcome. Multilevel linear regression.

^c^E-health4Uth and consultation vs control group: analyses were adjusted for age and the baseline value of each outcome. Multilevel linear regression.

^d^A higher score indicates more mental health problems (SDQ range 0-40, YSR range 0-210).

^e^A higher score indicates a better health-related quality of life (range 0-100).

### Interaction Effects

Exploratory interaction analyses showed 3 statistically significant interactions between the dummy variables for intervention groups and the demographic factors. Ethnicity moderated the intervention effect of E-health4Uth on condom use and gender moderated the intervention effect of E-health4Uth on alcohol consumption and the intervention effect of E-health4Uth and consultation on drug use (Table 4). More specifically, adolescents of Dutch ethnicity in the E-health4Uth group were more likely to use condoms during intercourse at follow-up compared to adolescents of Dutch ethnicity in the control group (OR 3.59, 95% CI 1.71-7.55), whereas there was no significant effect of the intervention among adolescents of non-Dutch ethnicity (OR 0.25, 95% CI 0.02-2.49). Furthermore, boys in the E-health4Uth and consultation group were more likely to use drugs at follow-up compared to boys in the control group (OR 0.36, 95% CI 0.13-0.96), whereas there was no significant intervention effect among girls (OR 4.47, 95% CI 0.72-27.74). Among boys and girls in the E-health4Uth and consultation group, no significant intervention effect was found on alcohol consumption (boys: OR 0.68, 95% CI 0.40-1.15, girls: OR 1.35, 95% CI 0.76-2.38).

**Table 4:** Stratified analyses of intervention effects on health behavior for the various levels of the significant moderator variables.^a^

Outcome				E-health4Uth vs control group		E-health4Uth +consult vs control group	
				OR (95% CI)^b^	*P* value	OR (95% CI)^c^	*P* value
**Alcohol consumption**							
	**Have been drunk or tipsy in the past 4 weeks**						
		**Gender**					
			Boys	0.68 (0.40, 1.15)	.15		
			Girls	1.35 (0.76, 2.38)	.31		
**Drugs use (past 4 weeks)**							
	**Gender**							
		Boys				0.36 (0.13, 0.96)	.04
		Girls				4.47 (0.72, 27.74)	.11
**Condom use during intercourse**							
	**Ethnicity**						
		Dutch		3.59 (1.71, 7.55)	.001		
		Non-Dutch		0.25 (0.03, 2.49)	.38		

^a^Only the results of the stratified analyses according to the significant moderators of the intervention effects are presented.

^b^Multilevel ordinal regression.

^c^Multilevel logistic regression.

## Discussion

### Principal Results

Using a cluster RCT, we evaluated the effect of E-health4Uth as a standalone intervention and in combination with an additional consultation for adolescents who were at risk of mental health problems. The E-health4Uth intervention as a standalone intervention showed minor positive results in a small number of outcomes, namely in the health-related quality of life and in condom use during intercourse among adolescents of Dutch ethnicity. The 2 positive results found in the E-health4Uth intervention were not replicated in the E-health4Uth and consultation group. The E-health4Uth and consultation intervention showed minor positive results in the mental health status of adolescents, but a negative effect on drug use among boys was found. In the subgroup of adolescents who were at risk of mental health problems at baseline and were, therefore, referred for a consultation with the nurse, the E-health4Uth and consultation group showed small to moderate positive results on mental health status and health-related quality of life at follow-up compared to adolescents in the control group who were at risk of mental health problems at baseline.

### Interpretation

Although it is promising that positive effects were found in the E-health4Uth group, only a small number of outcome measures were statistically significant (ie, health-related quality of life and condom use during intercourse), the effects were small, and the effects on condom use were only found among adolescents of Dutch ethnicity. Furthermore, because the E-health4Uth and consultation group received the same messages as the E-health4Uth group plus an additional consultation for the adolescents at risk of mental health problems, one would expect that the effects on condom use and health-related quality of life would have also been present in the E-health4Uth and consultation group. Although these effects pointed in the same direction, they were not significant in the E-health4Uth and consultation group. Therefore, the effects found in the E-health4Uth group have to be interpreted with caution.

In contrast to our hypothesis, we could not demonstrate that the E-health4Uth intervention was effective in promoting other health behaviors or the mental health status of adolescents. Although various studies show that Web-based tailoring is a promising technique to promote health behaviors and mental health status of adolescents [26-34], most studies are focused on older adolescents. Furthermore, the results of the evaluation of the appreciation of the tailored messages used in this trial showed that adolescents did not evaluate the tailored messages as explicitly positive in terms of their personally relevance [15]. If messages are not deemed personally relevant, the positive effect of these messages may be reduced [35]. Therefore, the tailored messages used in this study could potentially be improved further, possible resulting in messages that are more personally relevant and effective. The current messages could be further tailored by using, for example, demographics, personal cognitive factors (eg, manner in which health risks are perceived by the individual), social factors (eg, susceptibility to peer pressure), or the self-efficacy of the individual (eg, judgment of capability to change unhealthy behavior) [36,37]. Furthermore, algorithms generating tailored information can be easily extended to use more characteristics of the adolescent to tailor the messages, whereas wide-scale distribution can be arranged at relatively low cost.

Moreover, knowledge on how adolescents process and respond to personalized feedback is currently scarce [38]. More insight into how adolescents process the feedback messages, single messages, and when receiving multiple feedback messages on various behaviors at one point in time is needed to be able to improve interventions. Although the focus on multiple behaviors is becoming an increasingly popular strategy in interventions using Web-based tailored messages [39-41], adolescents receive a lot of information at the same time and it is conceivable that adolescents become overwhelmed by the amount of information. Furthermore, tailored messages were used and appreciated positively by adolescents in this trial [15] and they seemed interested in receiving feedback on health behaviors. In contrast to older people who are confronted with chronic diseases more often, adolescents are probably less likely to see the benefits of health behavior changes and consequently less likely to be internally motivated to invest time in health behavior changes [42].

As hypothesized, the E-health4Uth and consultation intervention was effective in enhancing the mental health status of adolescents. Furthermore, it is promising that expanding the Web-based tailored intervention with a consultation in the subgroup of adolescents at risk of mental health problems, improved the effectiveness of the intervention on mental health and health-related quality of life among these adolescents. The effect of the E-health4Uth and consultation intervention on the well-being of adolescents at risk of mental health problems was minor to moderate, in-line with the results of previous studies in which adolescents at risk of depression and anxiety, 2 components of the broader construct of mental health, benefited from an Internet program combined with a consultation [43-46]. A potential explanation for the effects on the well-being of adolescents is the dual approach of advice and a consultation. This approach guaranteed a repetition of the main mental health message and combined digital and oral feedback. However, it is also feasible that the consultation was responsible for the positive effects that were found and that the E-health4Uth questionnaire was primarily a useful way to select adolescents who needed further face-to-face support.

Because the nurses rated the information they received about the adolescents before the consultation as helpful in most cases (80.0%) [15], this information on adolescents’ health may have supported the nurse during the consultation to better tailor the information provided to the adolescent’s needs, thereby enhancing the effectiveness.

In contrast to our hypothesis, positive effects in the E-health4Uth and consultation group were not found in promoting health behaviors. Therefore, it might also be beneficial to apply the dual approach of advice and a consultation to the health behavior messages (ie, expand the Web-based tailored messages on the health behaviors with a consultation focused on these health behaviors), instead of primarily focusing on mental health in the consultation. A previous study, integrating Web-based tailored messages on fruit and vegetable intake with a consultation focused on this intake among schoolchildren showed promising results in the area of preventive youth health care [35]. However, future research is needed to investigate the degree to which the impact of Web-based tailored messages on health behaviors may be enhanced through expanding these messages with a consultation. Especially since an unexpected negative effect on drug use among boys was found in the E-health4Uth and consultation group. Although this result could be a random effect, another possible explanation is that giving information about drug use to adolescents raises adolescents’ curiosity about trying drugs. In earlier studies, a similar negative effect on drug use among Dutch adolescents was found [47,48]. In one of these studies, it was found that this increase in frequency was only a temporary effect [48]. However, it is an indication that one has to be careful with health promotion on drug use among adolescents and it highlights the importance of careful evaluation and in-depth study of how health promotion on drug use works for adolescents.

Because the Web-based tailored messages and the additional consultation were already interwoven with the existing practice of preventive youth health care, they are especially promising for future implementation. Implementing the Web-based tailored messages as a universal program (ie, offering it to all adolescents in a school class regardless of current symptom level or risk status) has multiple benefits. Universal programs, instead of programs that only focus on adolescents who are at risk (eg, for mental health problems), are often preferred by school administrators [49]. Additionally, by collecting information on the health of all adolescents in a school class, this approach presents an opportunity to select vulnerable adolescents and to enhance the efficiency of face-to-face consultations [7,12,13]. Efficiency is essential given the current financial strain on preventive health care.

### Strengths and Limitations

Important strengths of this study are the randomized controlled design and large sample size. The response rate was relatively high and our study population resembles the average Dutch adolescent population in secondary schools for gender, ethnicity, and education level [50]. However, this study was conducted only among Dutch adolescents aged 15-16 years in a preventive care setting; therefore, generalization to other countries, age groups, and settings should be done with caution. Furthermore, dropout was higher among girls, older adolescents, adolescents with a low education level, adolescents of non-Dutch ethnicity, and adolescents allocated to the control group instead of the E-health4Uth and consultation group, which could also affect the generalizability of the results. Nevertheless, we expect that the effects of our study would have been stronger without this selective dropout. A vulnerable group research has shown to be at a particularly heightened risk of mental health problems [15] and of exhibiting unhealthy behavior [1,51], dropped out while interventions are especially effective in high risk groups [52].

Additionally, the use of self-report measures may have resulted in less reliable outcomes. Therefore, the collection of more objective data on health behavior and additional parent and teacher ratings on the well-being of the adolescents may have been useful. Nevertheless, research suggests, for example, that self-reported alcohol consumption among adolescents is generally considered valid [53] and that adolescents are better reporters of their own mental health status than parents and teachers [54]. The percentages of adolescents with unhealthy behaviors and mental health problems in this study are largely comparable with the percentages of adolescents aged 15-16 years with unhealthy behaviors and mental health problems in the Netherlands [1]. Another limitation is that some adolescents in the control group may have received information from friends in the intervention groups despite the randomization of school classes. This may have contaminated the results. Furthermore, the overlap between the 2 intervention groups and control group is a limitation. In the E-health4Uth and consultation group, only adolescents at risk of mental health problems were invited for a consultation; thus, the other adolescents in this group received actually only the E-health4Uth intervention. Moreover, adolescents in all the groups could ask for a self-referral with the school nurse. Although only a few adolescents in the E-health4Uth group (17 of 533) and the control group (13 of 615) attended a consultation with the nurse (Figure 1), this may have underestimated the results. Therefore, in addition to the intention-to-treat analyses in which we analyzed adolescents in the groups to which they were randomized, regardless of whether they received the allocated intervention or not (eg, whether they attended the consultation or not after an invitation), we conducted exploratory per-protocol analyses. For these exploratory analyses, adolescents were allocated to the intervention they actually received (eg, the adolescents who self-referred to the nurse for a consultation were included in the E-health4Uth and consultation group for analysis purposes). The results from these per-protocol analyses were stronger than the results from the intention-to-treat analyses. That is, these analyses showed larger effects on mental health and health-related quality of life for the subgroup of adolescents at risk of mental health problems at baseline than the intention-to-treat analyses, suggesting that the results presented in this study may be underestimations of the actual effects. Unfortunately, information about the percentages of adolescents invited for a further consultation with the nurse or who were referred to another professional was not available because this information was not consistently administered by the nurse. However, the available data suggest that a low percentage of adolescents were invited for a further consultation with the nurse or referred to another professional. Further research is necessary to assess whether the consultation is an effective way in selecting adolescents who need help and providing them with the help they need.

### Conclusions

Findings from this study support the use of the E-health4Uth and consultation intervention in promoting the well-being of adolescents at risk of mental health problems. Compared to care as usual, E-health4Uth combined with a consultation was effective in promoting the mental health status and health-related quality of life in the subgroup of adolescents at risk of mental health problems. It is feasible that the consultation (and not the dual approach) was primarily responsible for these positive effects. However, E-health4Uth may have been a valuable tool to select vulnerable adolescents and to provide the nurse with information about the health of these adolescents. This could have contributed to the efficiency of the face-to-face consultation. Because the E-health4Uth and consultation intervention can be embedded in the existing practice of preventive youth health care, this increases the chance of future implementation. However, more research is needed to further evaluate the effects of the consultation as a standalone intervention and of the dual approach of further tailored eHealth messages and a consultation. Adding a consultation for adolescents at risk of mental health problems seems promising; therefore, future research is recommended to evaluate the potential effect of a consultation for adolescents who exhibit unhealthy behavior.

## Multimedia Appendix 1

Topics of the E-health4Uth modules.

## Multimedia Appendix 2

CONSORT-EHEALTH checklist V1.6.2 [55].

## Abbreviations

CHQ-CF-GH4: Child Health Questionnaire-Child Form-General Health
RCT: randomized controlled trial
SDQ: Strengths and Difficulties Questionnaire
YSR: Youth Self Report
