# Supplementary material for: Effectiveness of a Web-Based Tailored Intervention (E-health4Uth) and Consultation to Promote Adolescents’ Health: Randomized Controlled Trial
Source: J Med Internet Res. 2014 May 30;16(5):e143. doi: 10.2196/jmir.3163 (PMC4060146; doi:10.2196/jmir.3163)
Supplement: Supplementary file 1 [file jmir_v16i5e143_app1.pdf]

Appendix 1: Topics of the E-health modules

Behaviour and well-being	Items
Alcohol consumption	How often and how much the adolescent drinks alcohol (9 items)
Drug use	How often the adolescents has used different types of drugs (17 items)
Smoking	How often the adolescent smokes (2 items)
Sexual behaviour	How often the adolescent uses condoms during sexual intercourse (2 items)
Bullying	How often the adolescent is bullied at school, somewhere else or on the internet (3 items)
Mental health status	Strength and Difficulties Questionnaire (SDQ) (25 items) with a total score range 0 - 40
Suicidal thoughts	If the adolescent has had suicidal thoughts last year (1 item)
Suicide attempts	If the adolescent made a suicide attempt last year (1 item)
Unpleasant sexual experience	If the adolescent has ever had an unpleasant sexual experience (1 item)
